# A Reply to Some Remarks Contained in a Critical Analysis of Mr. Charles Bell's Paper on the Nerves of the Face

**Published:** 1830-01

**Authors:** 


### *A Reply to some Remarks contained in a Critical Analysis of* Mr. Charles Bell's *Paper on the Nerves of the Face.*

[We publish the following letter, because, from the quarter whence we received it, and the time employed in its composition, we suppose it to be as good an answer as can be made to the critical review of the modern discoveries on the Nerves, contained in our last Number. We expected, from the confident tone in which the writer pledged himself to us to give a complete refutation of our statements, that we should have been led, from the new light that would break in upon us, to make a manly and candid admission that our criticism was unfair and erroneous; that we had censured Mr. Bell unjustly, and attributed undeserved credit to M. Magendie and Mr. Mayo. Although we were conscious of having diligently striven to master the question, and to arbitrate fairly upon the points in dispute, some important evidence, we concluded, must have escaped our notice. We looked for nothing less than a strong, and clear, and decisive demonstration of our mistakes, and of the justness of those pretensions which we had considered unfounded. Something interested in a subject to which we had devoted many hours of patient attention, we were not incurious to have the means of thoroughly sifting it afforded us by the advocate of views opposed to our own, and we looked forward almost with anxiety to their production. Truth, we knew, is simple and clear, and easily intelligible; and, as *we* had given a definite shape and purport to *our* allegations, we anticipated from our correspondent a perspicuous, well-arranged, and decisive reply. We have been disappointed. Yet the letter, which follows, may possibly appear more satisfactory to others than to ourselves. We will not, by any further prefatory remarks, interfere to diminish the impression which its first perusal may make. Considering, however, that all our readers may not have studied this question as diligently as ourselves, we *subjoin* a few observations upon passages in the letter, which satisfied *us* of its real value, and induced us not to undertake any fuller analysis of it.—Editor.]

---

*To the Editor of the London Medical and Physical Journal.*

Sir: I am desirous of addressing some remarks to you on a review which has appeared in your last Journal, entitled "A Critical Analysis of Mr. Charles Bell's Paper on the Nerves of the Face." The influence which you possess, by conducting a Journal so extensively circulated, must make you anxious to exercise it with impartiality. I have to present views that vary essentially from those contained in the Critical Analysis alluded to, yet I hope you will insert them in the succeeding Number, that your readers may have a fair opportunity of judging of the real state of the question.

The writer has dwelt very briefly upon the paper which was the object of his analysis, but has devoted most of his attention to presenting a history of the discoveries which have been made in the nervous system, professing to explain not merely *what* has been done, but to *whom* the credit of each discovery is attributable. I find it an invidious task to follow him in this review, yet it is my duty to show the misconceptions which he has fallen into. He has placed before us, in a conspicuous light, a fact which demands particular attention: it is this, that, in the course of Mr. Bell's writings, he does not mention Mr. Herbert Mayo's name. The reviewer has attributed to Mr. Mayo what appear to be very important corrections of Mr. Bell's views; and he expresses his regret, also, that Mr. Bell altered his opinions to correspond with those corrections, without acknowledging to whom he was indebted.

I must remark, on entering upon this subject, that if, while showing that there was no just reason for Mr. Bell making any acknowledgment, I am constrained to refer to things which are unpleasant, the blame ought not to rest upon me. I myself am sorry that the writer of the review has revived this subject. There are reasons for believing that Mr. Mayo himself did not wish to continue the discussion.*

Let me now attend to what is assumed on the part of Mr. Mayo. He claims the merit of having repeated Mr. Bell's experiments, and shown the true nature of the 5th pair and of the portio dura. If there be any other things, these at least are the most important, and to them I beg to call the reader's attention.

It is of the first importance to attend to the manner in which Mr. Bell's opinions are represented. Let me examine, then, what is said of the 5th nerve. In Mr. Mayo's book (the Outlines of Physiology, p. 263,) it is said, "The branches of the latter nerve (the 5th) which emerge from the *frontal*, *infra-orbital*, and *mental foramina*, to supply the muscles and integuments of the face, Mr. Bell supposed

* See the letters in the Medical Gazette, in July 1828, and June 1829.

to be nerves *both* of *sensation* and *voluntary* motion." The reviewer, following Mr. Mayo, gives this account: "The muscles of the face, that is to say, the *nasal*, *labial*, and *palpebral* muscles, receive nerves from two sources, viz. the 5th cerebral nerve and the 7th. Now, of the two sets of nerves distributed to the muscles of the face, one, namely the 5th, had been long observed to resemble, in its mode of origin, the spinal nerves. It was probable, therefore, that the 5th serves in the face the same purpose as the spinal nerves in other parts. But the spinal nerves in other parts minister equally to sensation and motion: hence it followed that the *facial* branches of the 5th are probably nerves of sense and voluntary motion jointly." (P. 527.)

I can only say, in regard to these two quotations, that they do not represent Mr. Bell's opinions of the 5th; they are not given in his words; they are opposed to his words; they are inconsistent with his experiments on this nerve; they are inconsistent with his classification: however nearly they may be supposed to approach to his real views, they contain an error which is of paramount importance, as will be seen hereafter. *Mr. Bell never supposed that the influence of the 5th pair, as a muscular nerve, extended to any other parts than to those engaged in mastication.* To this I must beg particular attention, because, on every occasion when Mr. Mayo's pretensions have been placed before the public, this true representation of Mr. Bell's opinion of the 5th pair, as a muscular nerve, has not been given, but the other account has been substituted. Mr. Bell applies to the 5th, in the most distinct way, the name of "the nerve of sensation and mastication;" and he is particular in limiting its functions as a motor nerve to the jaws and lips. It will be seen hereafter, indeed, that the circumstance of its being confined solely to these parts is the principal argument for the arrangement of the nerves into the "symmetrical" and "irregular" nerves.

I will now make extracts from Mr. Bell's first paper delivered to the Royal Society. But I must first mention that there is an inaccuracy in it, which has been explained in the last paper. Mr. Bell was of opinion that the lips had a power of motion in combination with the actions of chewing, and he was led to attribute this to the influence of the infra-orbital branch of the 5th. It has been shown that the infra-orbital is solely a nerve of sensation, and that this combination of the lips and cheeks with the motion of the jaws depends on another nerve, the labio-buccinalis. This, as it will be seen, makes no difference in the proofs which I have to bring, that it was *those parts of the face only which are engaged in mastication that were supposed to receive any muscular power from the 5th nerve.*

While giving a short comparative view of the 5th, in introduction to the account of his system, Mr. Bell says, "From the nerve that comes off from the anterior ganglion of the leech, and which supplies the mouth, we may trace up through the gradations of animals a nerve of *taste and manducation*, until we arrive at the complete distribution of the 5th, or trigeminus, in man." (P. 14.) "We have seen that when the 5th nerve, the *nerve of mastication and sensation*, was cut in an ass, the animal could no longer gather his food. In the individual whose face was paralysed on one side (by disease of the portio dura), during the excited state of the respiratory organs, there could be observed no debility or paralysis in the same muscles *when he took a morsel in his mouth, and began to chew.*" But the most remarkable and convincing evidence of his considering the *facial* branches to be compound only as regards the lips, is to be found in the account which he gives of his experiments on the infra-orbital nerve. This nerve, it is well known, is distributed profusely to the muscles which operate on the cartilages of the nose, and to those which move the lips. It is expressly stated that, when the nerve was divided, "*no change took place in the motion of the nostril; the cartilages continued to expand regularly in time with the other parts which combine in the act of respiration, but the side of the lip was observed to hang low, and it was dragged to the other side.*" By this exception, made, it must be observed, under a wrong notion of this branch being a compound nerve, we have the best evidence of the limited function which he supposed the 5th nerve possessed. But what does he say of the frontal branch, which it is asserted was supposed by Mr. Bell to be a nerve of voluntary motion and sensation. This is a nerve far removed from the parts engaged in chewing. Accordingly, we find that Mr. Bell has been at the pains to prove that it has no power of influencing the muscles. "I divided the branch of the 5th pair which goes to the forehead, in a man, at his urgent request, on account of the tic douloureux: there followed no paralysis of the muscles of the eyebrow; but in an individual where an ulcer and abscess, seated anterior to the tube of the ear, affected the superior branch of the respiratory nerve, the eyebrow fell low, and did not follow the other when the features were animated by discourse or emotion." (P. 23.) Mr. Mayo has said that he corrected Mr. Bell for saying that this branch of the 5th was a nerve of voluntary motion and sensation, yet he has never related any experiment to prove whether that opinion was correct or not! The only reference which he ever makes to this nerve is in giving a quotation of the above sentence in the "Commentaries," to which he refers us, (see No. i. p. 118.) This, among other things, proves that his present opinions of Mr. Bell's paper are inconsistent with what he entertained at that time.

Let us next take the portio dura, to examine whether Mr. Mayo has correctly represented the opinions which Mr. Bell entertained of it.

It is not to be wondered at, that, having been mistaken with regard to the facial branches of the 5th, he should fail to represent what were Mr. Bell's true opinions of the other nerve that goes to the same parts, viz. the portio dura. However well the functions which he attributes to this nerve chime in or correspond with those which he gives of the 5th, the account is quite erroneous. Mr. Mayo says (at p. 263, Outlines of Physiology,) "Mr. Bell thought that he had obtained by this experiment a proof that the portio dura controls the *instinctive actions, but not the voluntary actions, of the muscles of the face.*" Again, at p. 333, "In other words, according to Mr. Bell, the 7th is the *nerve of instinctive motion* to the face, and the 5th of voluntary motion and sensation." The reviewer, also, following Mr. Mayo, has these words: "Mr. Bell concluded that the portio dura of the 7th (the superadded or respiratory nerve of the face, as he termed it,) was placed for the purpose of transmitting the *instinctive impulse* to muscles already supplied with sentient and voluntary nerves from other sources, namely, from the *facial* branches of the 5th." (P. 527.)

Your readers must already have seen, even from those experiments which have been alluded to, that it was *impossible* Mr. Bell could hold the opinion which is attributed to him. How often has it been repeated, when these same statements were made before, that Mr. Bell never once used the word "instinctive" in any of his writings upon the nerves: it must surely, then, be inconsistent with his opinions; it is a word thrust upon him by Mr. Mayo. The writer of the review, believing that the word instinctive is a fit word, contends against Mr. Bell in this manner: "The whole frame is susceptible, although in a lesser degree, of instinctive excitation, as well as the countenance and throat: the *muscles of the jaws,* especially, which have almost as much to do in instinctive action as the muscles of the face, have no superadded nerve given to them." I allow this. The chicken, newly hatched, can pick the corn by instinct, just as it can stand or run. It is most probable that this very consideration, that there was no distinction to be drawn, by the application of the principle of instinct, between the muscles of mastication and those of the face, prevented Mr. Bell using the word instinct. For we may learn that it was, in fact, by contrasting these muscles of the jaws with those of the face, that he was led to call the portio dura the *respiratory* nerve of the face.

The account which has been given of this nerve, as it stands opposed to Mr. Bell's words, is contrary both to the experiments which he relates and to the instances which he gives in illustration of the functions of this nerve. When the infra-orbital branch of the 5th was divided, in an experiment upon an ass, we have seen that he pointedly stated that "no change took place in the motions of the nostril." When the frontal branch of the 5th was divided, there followed no paralysis of the muscles of the eyebrow. These parts must, therefore, depend exclusively for the motion which they possess upon the portio dura, the remaining nerve. Accordingly, it is said, in another place, when "the portio dura was divided on one side of the head, the motion of the nostril of the same side instantly ceased." Again, when the branches of the portio dura going to the forehead were destroyed by disease, "the eyebrow fell low, and did not follow the other when the features were animated by discourse or emotion." This nerve (the portio dura) Mr. Bell concluded to be the only nerve of motion to all the parts of the face, except those which are engaged in mastication. It was in regard to the lips alone that there was any exception made. How, then, could it be said that it was the nerve of instinct only?

But it is further alleged, that he considered the portio dura to be an *involuntary* nerve; that it controlled "those movements of the features which are usually called instinctive, as opposed to such as are premeditated." Mr. Bell has not characterised this nerve either as a voluntary or involuntary nerve. Whatever the word instinctive, applied to this nerve, may be thought to indicate, the word "respiratory," which Mr. Bell has used, has, according to his explanation, no reference to these questions. From the examples which are given, it is obvious that he knew, at the beginning, that its functions were not either altogether voluntary or involuntary; that it partook of the two pro-

2

perties: and this opinion he holds still, however others may conceive they have improved our knowledge by saying it is exclusively voluntary. Although the function of breathing generally is carried on without the superintendence of our volition, yet it is necessarily placed in some degree, for the purposes of speech especially, under our control. The examples which Mr. Bell has given of apoplexy and of sleep prove that this nerve, the portio dura, influences the muscles independently of volition; and the other examples, such as those of *speaking*, *whistling*, *singing*, &c., sufficiently prove it can bring the muscles under the direct control of the will.

I repeat, that the experiments which Mr. Bell made on the 5th pair, those on the portio dura itself, and the observations and remarks connected with them, are all broadly opposed to the view which has been represented as his opinion. It must appear inexplicable, since it is the correction of errors only which has been the cause of the continuance of this contest about the facial branches of the 5th and the portio dura, why Mr. Mayo never mentions Mr. Bell's opinions in his own words; why he does not allow at once that Mr. Bell called the 5th nerve "the nerve of mastication and sensation." The reviewer states what he conceives were the notions entertained by Mr. Bell, but he also avoids using Mr. Bell's words. This is a carelessness in conducting a review of these discoveries which ought surely to be condemned; for behold what it has led to. It has led Mr. Mayo to suppose that it was he himself who first suggested what is the true nature of this nerve. He claims to have discovered that this is the nerve of motion to the muscles of the jaws and the nerve of sensation.*

The same series of misconceptions, and the same negligence of ascertaining the true opinions of Mr. Bell, have made Mr. Mayo believe that he has destroyed the whole system proposed by that gentleman.† The reviewer says, "And these conclusions of Mr. Mayo's, Mr. Bell has subsequently published as his own, having, as it appears to us, substantially abandoned his theory of the respiratory nerves, and adopted a truer view, without (we regret to say) acknowledging to whom he is indebted for the correction." All that I need remark is, that Mr. Bell has not departed from the opinions which he has expressed in the first or introductory paper, presented in 1821 to the Royal Society.

* See No. ii. Anat. and Phys. Commentaries, and Outlines, p. 336.
† See Outlines, p. 333.

And it may be already seen that Mr. Mayo has not effected what he says, *because Mr. Bell never entertained the views which have been attributed to them.* If Mr. Mayo shall again aim at correcting this system, let him represent it in a manner that we may recognise it: hitherto he has only wasted his shafts by shooting at something of his own creation.

We have a very convincing proof that it is not from him that we can at present take the representation of Mr. Bell's system or classification. He conceives that he has discomfited Mr. Bell's arrangement of the nerves, and yet it is singular that he does not even allude, in the whole course of his work, to the main and principal class, viz. that of the spinal nerves and the 5th pair! There is not a single hint of Mr. Bell having held any opinions respecting the nature and functions of these nerves.

It is respect for the character of your Journal which alone makes me proceed with this subject.

It is well known to every one who has attended to the history of these discoveries, that the opinions which Mr. Bell holds concerning the superadded system had their origin in those which he entertained of the regular or symmetrical system: that is, the spinal nerves and 5th pair. It was only when he had ascertained what their precise functions were, that he was enabled to investigate the others. Taking the nerves of the face, for example, he said that the 5th pair resembled the spinal nerves; that is, it has two roots, and it consequently has two functions: it has motion and sensibility. But he observed that it was limited as a motor nerve to those actions only which are connected with chewing and feeding. There is another nerve in the face, viz. the portio dura: this does not interfere with the motions of chewing, but commands exclusively a different class of muscles. The question to be resolved, therefore, is, why should the muscular part of the 5th be confined to mastication, and why should the portio dura be confined to the muscles of the face? Why should not a spinal nerve be all sufficient, just as in the arm or leg, to control both the jaws and the features? Every person accustomed to look upon the framework of the human body as affording proofs of design, must grant that there is here a difficulty to be resolved. I do not presume to express any opinion as to the justness of Mr. Bell's explanation, and I do not choose to attempt, within these short limits, to present what I conceive to be his views. But it can be seen, from the beginning, that he considered the actions of chewing were different from those which characterised the features. The 5th, being one of the symmetrical system, he conceived to be a nerve "common" to all animals that have locomotion and sensation, and which require to grasp, to feel, and to consume their food. It is a nerve which is found in all creatures, whether they have a concentrated apparatus for breathing or not. We may see that he even thought that a ganglion and nerves analogous to the 5th might be recognised in the vermes, supplying their mouth. The portio dura he included in a different class of nerves from the 5th. He seems to have conceived that, when respiration by lungs was added to the original simple structure of animals, the nervous system underwent a corresponding change. This complicated organ of respiration by lungs makes new nerves be required, to combine many parts that are distant with the motions of the lungs. And the portio dura, he conceived, arose from a particular part in the brain, in combination with other nerves, to connect the features, and especially the nostrils, with the motion of the lungs.

No friend to science can object to the general doctrine proposed by Mr. Bell being criticised: it was to be expected that differences of opinion would arise: but let not the facts be disturbed; let us acknowledge that all these have been correctly stated. If any other doctrine be proposed, it must embrace the explanation of these facts. When representing his general doctrine, it is common courtesy that his words should not be changed.

Your readers will join with me in expressing some wonder that the claims of Mr. Mayo should require discussion at this late period of time; for it is nearly nine years since Mr. Bell presented his first paper to the Royal Society. The cause of the delay is to be found by referring to Mr. Mayo's method of proceeding. He commenced his connexion with this subject by writing a review of that first paper. His criticism was of the most sweeping and condemning nature. Nothing escaped, except one point, viz. an experiment on the infra-orbital nerve, which, it is remarkable, Magendie (who repeated Mr. Bell's experiment) had previously shown to be inaccurate, and which Mr. Bell himself has allowed, in his last paper, to be the only thing which has been corrected. With regard to this experiment, Mr. Mayo has said that the inference which Mr. Bell drew from it was the only correct thing in the whole paper; but he adds, it is only an "experimental confirmation of an opinion which, at the beginning of the eighteenth century, occurred to Dr. Blair, on his minute examination of the proboscis of an elephant, viz. that the infra-orbital nerves are *nerves of touch.*" Having written this review, Mr. Mayo proceeded to publish on the Nerves, but he avoided referring to Mr. Bell's opinions. His second paper was on the functions of the portio dura, the 5th pair, the spinal nerves, and the resemblance of the 5th pair to the spinal nerves. From this paper no one could discover that Mr. Bell had been engaged on these subjects at all. It was not till he published the "Outlines of Physiology," in this year, 1829, that he again returned to the attack on Mr. Bell's system, and explained how it was he had proved it to be erroneous.

It becomes necessary for me to consider what were some of the remarkable circumstances contained in that paper which Mr. Mayo criticised. It is not, as the reviewer represents, a paper on the "superadded or respiratory nerves," but it is "an account of some experiments on the nerves, which lead to a new arrangement of the system." It contains an arrangement of all the nerves of the body. It is principally occupied in contrasting the symmetrical system of nerves with the respiratory system. There are examples given of both these classes, the one as the 5th pair, the other as the 7th; and these stand, as it were, the representatives of the two great classes.

Yet it is not from what is said concerning the particular functions of each individual nerve that the paper derives its chief interest. It is the *new principle of investigating the nerves* which is there proposed. Mr. Bell announced, for the first time, that nerves are not all equally capable of bestowing the same powers, but that each nerve had its own appropriate function to perform. He cut across one nerve, and the sensibility was destroyed, although another nerve remained to supply the same part; he cut across a second nerve, and, although the sensibility remained entire, the muscles were rendered completely paralytic. He said, "No organ which possesses only one property or endowment has more than one nerve, however exquisite the sense or action may be; but, if two nerves, coming from different sources, are directed to one part, this is the sign of a double function performed by it." The nerves of the animal frame are complex in proportion to the variety of functions which the parts have to maintain." (P. 7.) It was this new principle which, in the eyes of all Europe, I may say, was regarded as the remarkable feature. The reviewer, however, has failed to notice this. It was this new principle, illustrated with examples, which created a new era in the investigation of the nerves. After this, crowds of experimentalists, with scalpels in their hands, guided by this new principle, were joined together in the pursuit of distinct functions of the nerves.

But the classification of the nerves is another remarkable feature of the paper. In accordance with the new principle, he pointed out that all the nerves which have two roots have also two separate functions; and he classed together the 5th pair and the spinal nerves, as all possessing the same characters. As to those nerves which have only single roots, he separated them into another class, and showed that they possessed distinct and appropriate powers. If we be led to inquire what the experiments were which authorised him to announce this principle and this classification in the first paper, we shall find that it was not from those few experiments which are related that he could have arrived at his conclusions. All who are acquainted with the subject know that he had made numerous experiments before presenting this paper; and perhaps the most remarkable were those which he made on the roots of the spinal nerves in 1809, and in March 1821.

To this first paper, then, with its principles and classification, Mr. Mayo was hostile from the beginning. He took the par vagum, and, by his experiments on it, seemed to wish to cast a ridicule upon the subject. Objecting to the classification of those nerves which have two roots and a ganglion, (that is, of the 5th pair, with the spinal nerves, according to Mr. Bell,) he says, that the par vagum may be included among them. I do not deem it necessary to point out to anatomists that there is no correspondence in the roots and the ganglion (if it be one) of the par vagum with these parts in the spinal nerves and the 5th pair. But what analogy do his experiments prove to exist? Mr. Bell divided the 5th, and sensation was gone wherever that particular nerve was distributed. Mr. Mayo did not divide the par vagum across, but, to prove that it was the nerve of sensation, he *pinched* it, and "the animal gave violent indications of pain." He did not think it necessary to cut it across, and see whether the stomach, the lungs, the œsophagus, the parts to which it is distributed, lost their sensation!

I may now ask the question, if, after rejecting all that was laid down in Mr. Bell's first paper, Mr. Mayo ought to take advantage of the same principles, the same classification, and the very same facts, which are contained in it, without referring to the name of the discoverer? In the second paper which Mr. Mayo wrote, he selected the portio dura, the 5th pair, and the spinal nerves, to be the subjects of his investigation, and yet he does not once mention Mr. Bell's name.

The reviewer has said that Mr. Mayo proved that the "ganglionless" portion of the 5th pair is the nerve of motion; that this root is distinct in function from the one that has a ganglion upon it. It was Mr. Bell who first established that nerves which have double roots have two distinct functions. He first made the contrast between a nerve, such as the portio dura, which has only a single root and no ganglion upon it, with another, such as the 5th pair, which has two roots and a ganglion on one of them. It was he also who pointed out the importance of a ganglion, by showing that, in the 5th, for example, all the nerves proceeding from the ganglion over the whole head are endowed with sensibility. It was to him we owe the knowledge of the fact that certain branches of this nerve, limited to the jaws and lips, had motion combined with sensation. In explanation of this double character of the 5th, Mr. Bell said that its roots were double, like those of the spinal nerves; that it had a ganglion, and that "some filaments pass onwards without entering the ganglion." Now, it was upon these filaments which pass the ganglion that Mr. Bell conceived motion depended, although he may not have said so in "so many words," as the reviewer expresses it. Indeed, it will be found that the only satisfactory proofs by which the truth can be demonstrated, that the 5th is a motor nerve at all, were advanced by him.

We have seen that Mr. Mayo rejected the explanation of Mr. Bell, that nerves which have double roots are for motion and sensation: I must beg to remark, that it is only by depending upon the truth of this principle that his conclusion can be justified at all. The strict anatomy of the origin of this nerve by two roots, and the course of those filaments which pass the ganglion, were long ago well known to anatomists. To prove this, see the writings of Wrisberg, Santorini, Soemmering, Paletta, Prochaska, Meyer, Pfeffinger, Martin, Gunther, Haase, Vicq d'Azyr, and Scarpa, who described the anterior root mingling with the third division, and with it alone.* But it never occurred to any of these eminent anatomists, that the one root was exclusively the nerve of motion, and distinct from the other. Paletta made the conjecture, but none who followed

* See the last edition of Mr. Bell's System of Anatomy, vol. ii.

put faith in it: he thought that this root was a motor nerve to the muscles of the jaws and cheeks, just as the 3d is the motor to the muscles of the eye. The distribution of this part of the nerve to muscles alone could not, however, suggest a distinction between it and the ganglionic portion, as they are joined together, and the branches of the latter are as profusely supplied to the muscles of the face as those of the former to the jaws. It follows, then, that experiments were wanting. Mr. Bell proved, by direct experiments, that the filaments of the spinal nerve which pass the ganglion, that is, the filaments which correspond with the ganglionless part of the 5th, constitute that root which alone bestows the power of motion upon the spinal nerve. Again, having deduced from the anatomy that the 5th resembled the spinal nerves, experiments were made which proved that the third division of the 5th pair alone, of all this widely distributed nerve, bestowed motion. Now, it was after such proofs were supplied in the writings of Mr. Bell and of Mr. Shaw, that Mr. Mayo published his second paper. Yet he did not refer to the above experiments, either those upon the spinal nerves or those upon the third division of the 5th, however much they tended to illustrate his conjecture. This, it will be seen, places great restraint upon his readers, because the experiments upon all the branches of the 5th which he enumerates, (that is, four, or perhaps more, in number,) *prove that it is not in any respect a motor nerve.* In the experiments to which he refers, the branches of the 5th, which go profusely into the muscles, were proved to possess *no* power of drawing the muscles into action. The 5th could only be known, by the experiments referred to, to be a nerve of sensation, and nothing more.

The remarkable difference between Mr. Bell's method of proceeding, and that which Mr. Mayo pursued, in investigating this point, consisted, as your readers may have already seen, in this: Mr. Bell made his experiments on the roots of the spinal nerves before directing his attention to the 5th pair; while, on the contrary, Mr. Mayo went to the 5th at once, and then was directed to the spinal nerves.* It was by judging from the analogy between the 5th pair and the nerves of the spine that Mr. Bell was prepared to attribute to the 5th similar functions to those possessed by the spinal nerves. To prove that he had made experiments on the roots of the spinal nerves, I may refer to a paper by

* See No. ii. Anat. and Phys. Comment., Outlines, &c.

Mr. Shaw, read before the Medico-Chirurgical Society, in April 1822, in which the first experiments by Mr. Bell (those performed in 1809) are described; and to another paper published in this Journal in October 1822, in which he describes experiments on the spinal nerves, which were made publicly in the School of Great Windmill street, in March 1821. Both of these series of experiments contain convincing proofs of the anterior "ganglionless" portion of the spinal nerve being for motion. Mr. Mayo pursued an opposite course, I have said: he first directed his attention to the 5th pair, and then he formed his conjectures of the spinal nerves. He says that, reflecting on the analogy of the roots of the spinal nerves with those of the 5th pair, as it has been observed by Soemmering, he was led to think that the one root of the spinal nerve belonged to motion, and the other to sensation. "When I was engaged in experiments to determine the fact, M. Magendie's were published, which establish the justness of my conjecture."* But this paper was not published till July 1823.

I have said that Mr. Bell was engaged in experiments upon the spinal nerves before he made his experiments on the 5th pair; and I have said that he deduced his opinions concerning the functions of the 5th pair from these experiments. Now, this will not appear from the account which Mr. Mayo has given of the result of the experiments made by Mr. Bell: no more will it appear from what the reviewer has said. I had formerly to complain that they did not make use of Mr. Bell's own expressions, in pointing out his opinion of the 5th pair and of the portio dura: now I have to complain that they make extracts from an essay which was *never published*, and fix Mr. Bell's opinions of the spinal nerves to what he then said, more than twenty years ago. They seem to regard as of no consequence those opinions of the spinal nerves which he gives in his first *published* paper. This has led them both into great inconsistences. The reviewer confounds the reader by making two references, one to the unpublished pamphlet of 1809, and another to the paper in the Philosophical Transactions of 1821. He does not seem aware either of the injustice or of the contradiction which is consequent upon this; inasmuch as in the first he gives *ten or a dozen* functions to the spinal nerves, and in the latter only *two*. It is for the purpose of showing how incorrect Mr. Bell's experiments on the roots of the spinal nerves were, that he

* Anat. and Phys. Comment. No. ii. p. 10.

refers to the unpublished paper, and says, that he considered "that the anterior roots, or fasciculi, of the spinal nerves were the organs of consciousness, of thought, feeling, and volition; and that the posterior roots regulate the secret, organic, or automatic functions of the body, secretion, growth, the sympathies of the parts, and the like." When he refers, however, to Mr. Bell's first published paper, and speaks of the classification of the spinal nerves and 5th, he let us see, without any explanation, that these functions were reduced only to two, viz. *muscular power* and *sensation!* Not startled in any way by this contradiction, we find, in a subsequent part of his review, that he removes all the merit from Mr. Bell for his discoveries in the spinal nerves, and confers it upon M. Magendie, although the latter made no experiments upon these nerves until Mr. Bell's paper was more than a year before the public. "We attribute this great discovery (that is, of the distinct functions of the roots of the spinal nerves,) entirely to Magendie. It is childish in any other physiologist to advance a claim to it." The little essay which the reviewer has got into his possession, and from which he has made extracts, was, as he himself informs us, printed *twenty* years ago, and was *circulated among Mr. Bell's private friends.* It bears on its title that it was an "Idea of a new Anatomy of the Brain, submitted for the observations of his friends;" and it was never published. Having perused that essay, I do not feel inclined to grant that Mr. Bell did really entertain those opinions which are attributed to him, even at that time. Quotations might be given to prove that these opinions are inconsistent with many passages in this work. There are speculations, however, concerning the functions of the cerebrum and cerebellum, which, being connected with the experiments on the spinal nerves, may give rise to some dispute about what his real opinions were at that distant period. But let the statements of the observations made during the experiments be kept detached from these speculations, and we have presented to us a series of most important facts. It is needless, however, to discuss about the contents of this work: it was not written for the public; and when Mr. Bell published his views, if he entertained those erroneous notions, he must have afterwards rejected them. We have, indeed, his own authority for saying that he did abandon these investigations into the functions of the cerebrum and cerebellum, and that he confined himself solely to the nerves proceeding from them.* There is surely

* See a letter in the Medical Gazette, May 1829.

an impropriety, therefore, in extracting opinions from that work, and attributing them as the most approved which he has given, when they are found to be perfectly inconsistent with what he has *published.* The only use that can with fairness be made of this essay, is to show how the author has improved his opinions from the year 1809, rejecting what was bad, selecting what was good, and extending his knowledge of the system. It is strange that Mr. Mayo should believe that Mr. Bell always held these opinions about the spinal nerves possessing so many separate functions; for when he attempted, as we have seen, to prove that the par vagum was like one of these spinal nerves, he considered it sufficient, in order to establish the resemblance, to prove, by experiment, that it was only endowed with *motion* and *sensation.*

But that other experiments on the roots of the spinal nerves, besides those which are alluded to, were made during the interval, when the reviewer says "the subject slept for awhile," we may learn from your own Journal for October 1822. The experiments are described there which were performed in March 1821; that is, five months before the reading of Mr. Bell's first paper to the Royal Society. Now, the same objections cannot be applicable to them as to the former, since no mention whatever is made of the cerebrum and cerebellum. The notes of Mr. Shaw, together with those of one of the most intelligent of the pupils who assisted, prove that, "upon irritating the posterior roots of the spinal nerves in three or four places, no effect was produced upon the neighbouring muscles; but, when the anterior root singly, or the whole spinal nerve, was pinched by the forceps or pricked by the scissors, an evident motion was produced on the muscles, not only perceptible to the eye, but, when the third or fourth dorsal nerve was touched, the whole scapula moved in the hand of the assistant. This motion was not communicated to the muscles when the ganglion which is formed on the posterior root within the sheath was touched: neither did it follow an injury of the posterior column of the spinal marrow."

But, to close my evidence that there was no reason for Mr. Mayo or the reviewer fixing these erroneous opinions concerning the roots of the spinal nerves upon Mr. Bell, I may refer to the paper by Mr. Shaw, published in the Medico-Chirurgical Transactions, and read in April 1822. Although much is said in that paper of Mr. Bell's views of the spinal nerves, nothing can be found which at all coincides with the false opinions ascribed to him; but there is a great deal which proves how it was that he came to reduce the number of functions to two. I must, in particular, direct your readers to the last division of his paper, where the question is discussed, "Why *sensation* should remain entire in a limb when all *voluntary power* over the actions of its muscles is gone, or why *muscular power* should remain when *feeling* is gone." The explanation of these phenomena could not be more accurately given, even if the question were to be discussed in the present day. It may, perhaps, afford additional interest to this paper, to know that it was published not only before Mr. Mayo was about to make the discovery of the roots of the spinal nerves, but before Magendie's repetition of these experiments had been brought to this country.

But, even after reading these various accounts of experiments upon the spinal nerves, proper justice cannot be done to Mr. Bell, without at the same time showing what were the nature and object of his experiments on the 5th pair and the portio dura. His experiments upon the spinal nerves could not be considered complete until the 5th pair in the head, which has a ganglion upon the root corresponding with the posterior root of the spinal nerve, was shown to be the only nerve of sensation to the head. It is obvious that the experiments upon the roots of the spinal nerves must necessarily be attended with doubt, when the object is to ascertain the degree of sensibility possessed by the different roots. Mr. Bell has always said that he relied upon his experiments on the nerves of the face for establishing the true nature of the posterior roots which have ganglions upon them. As to the anterior "ganglionless" roots, these are the only ones of which he had formerly been perfectly sure. It is strange that, notwithstanding the care with which he has always limited the function of the anterior roots to motion, it is alleged that he believed *sensation* was one of its functions. It does not seem to be known that this opinion belongs only to M. Magendie. He, trusting exclusively to the experiments of breaking up the bones of the spine, and exposing the spinal marrow, and ignorant of the analogy of the 5th, came to this conclusion, that the anterior roots bestow the power of motion, and *are also endowed with sensibility to a certain degree* :* which conclusion, however, as it has been obtained by a method of inquiry apt to lead into errors, we may put little faith in. It tends only to show that the reviewer and Mr. Mayo have been

* See the last paper in the first vol. of his Experimental Physiology.

somewhat negligent in their endeavours to adjust exactly what belongs to Mr. Bell, and what belongs to the other physiologists who followed him in these important investigations.

I am your obedient servant,
AN OLD PUPIL OF WINDMILL STREET.

---

We should tax our readers' patience too severely, if we were to comment upon every part of the preceding prolix statement. It will be sufficient for our purpose to try what the thread of the "Old Pupil's" argument is made of, by straining it at a few points. We shall soon see whether it be of unsound materials or not.

1. "Mr. Bell," says the letter-writer, "was of opinion that the lips had a power of motion in combination with the actions of chewing, and he was led to attribute this to the influence of the infra-orbital branch of the 5th."—Why, this is one of the main points we have laboured to establish. This is exactly where we say that Mr. Mayo set Mr. Bell right. Mr. Mayo showed that the infra-orbital nerves have nothing to do with motion. He proved, in opposition to, and in correction of, Mr. Bell, that, as it regards the facial muscles, the portio dura is the exclusive motor nerve. If the reader wishes to know of how much importance this discovery of Mr. Mayo's then was, let him turn to vol. xii. part i. of the Medico-Chirurgical Transactions: he will there see the system of *false* pathology which grew out of Mr. Bell's theory expounded by the late Mr. Shaw; and cases stated (similar to one described by Mr. Bell,) in which, the portio dura being affected, motion was *seen to be lost in the facial muscles on those occasions only when they acted as respiratory muscles; to be perfect when they acted as chewing muscles!* When a patient, in whom the portio dura was affected, "blew, or attempted to whistle, the air escaped by the right angle of the mouth," &c. "*On exciting those actions, which, by experiments on animals, we had proved to depend on the 5th pair of nerves, they were all found perfect; i. e. the patient could bring the orbicularis oris into such action as to hold a pencil with it!*" &c.* In another case of a parallel description, "on observing the action of the muscles while the lady was eating, I did not perceive," said Mr. Shaw, "any power deficient; but the moment *she smiled or laughed,* there was distinct paralysis!"

* Medico-Chirurgical Trans. vol. xii. part i. p. 111.

2. "Mr. Mayo," the 'Old Pupil' observes, "has said that he corrected Mr. Bell for saying that this branch of the 5th (the frontal) was a nerve of voluntary motion and sensation; yet he has never related any experiments to prove whether that opinion be correct or not!"

We turn to Mr. Mayo's Anatomical Commentaries, part i. p. 110: "Some days after this, the *frontal nerve was divided,*" says Mr. Mayo, "on one side of the forehead of the same ass; when the neighbouring surface appeared to have lost sensation, but its muscles were not paralysed!"

We do not attribute to the "Old Pupil" deliberate misrepresentation in this instance. He probably supposed that Mr. Mayo had not made any such experiments as we have shown he had made. But we lay our finger upon this point, as one out of repeated instances in which the writer of the "Reply" endeavours to turn the reader's attention to some smaller question, and to divert us from the broad and general fact, that Mr. Mayo established *that all the facial and other ganglionic branches of the 5th are nerves of sense alone.*

3. We gave it as our opinion in the review which has drawn forth the foregoing "Reply," that it was Mr. Mayo who had ascertained the true nature and distribution of the ganglionless part of the 5th. Hear what Mr. Bell's advocate says in answer to this, in favor of that gentleman's claim to this discovery. "It was upon those filaments which pass (below) the ganglion that Mr. Bell conceived motion depended, *although he may not have said so 'in so many words,' as the reviewer expresses it.*" Indeed! Are we really to believe that, when Mr. Bell attributed *motive power* to a *ganglionic* part of the 5th, (namely, the infra-orbital nerve, in which every body knows, and knew, no ganglionless filament is involved,) that he had any notion that the *ganglionless part* of the 5th was the *exclusively motor portion.*

4. The "Old Pupil" asserts that "the reviewer confounds the reader by making two references, one to the unpublished pamphlet of 1809, and another to the paper in the Philosophical Transactions of 1821. He does not seem aware either of the injustice or of the contradiction which is consequent upon this, inasmuch as in the first he gives *ten or a dozen* functions to the spinal nerves, and in the latter only *two.*" Our respondent is mistaken: the contradiction exists only in *his statement* of our opinions. Mr. Bell, we asserted, and re-assert, in 1809 attributed to the agency of the spinal nerves sense and motion, and various automatic influences in addition. We have no where asserted that he has ceased to do this now. On the contrary, we believe that he continues in this persuasion. All other physiologists, at least, are of opinion that the spinal nerves exert this triple function, and consider them not merely as organs of consciousness, but likewise to exert an influence in growth, secretion, and the like. In 1809, Mr. Bell, as we have shown, supposed the anterior or ganglionic roots of the spinal nerves to be nerves both of sense and motion. His advocate, however, implies that, before M. Magendie's experiments were published, Mr. Bell had changed his first opinion, and adopted the belief that the ganglionless portions of the nerve were for motion alone, the ganglionic for sensation alone.

Now, in the first place, it is not very likely that Mr. Bell concluded that the ganglionic roots of the spinal nerves were for sense alone, at the time when he published his opinion, backed by experiment, that a large part of the ganglionic branch of the 5th (the infra-orbital) was for sense and motion jointly. The letter-writer adverts continually to Mr. Bell's parallel between the 5th and spinal nerves: that parallel had been drawn before, and most strikingly, by others. Yet Mr. Bell's adoption of it gives force to the preceding argument against his knowledge of the functions of the two roots of the spinal nerves antecedently to Magendie's experiments. Mr. Bell *strictly* compares together the 5th and the spinal nerves; and it would be a strange mode of arguing to deduce from his imperfect knowledge of the functions of the one, his perfect acquaintance with the functions of the other. Mr. Bell certainly did not publish any definite and correct ideas of the distinct functions of the anterior and posterior fasciculi of the spinal nerves before Magendie, and, to an unprejudiced mind, the following extract from the paper of 1821 must thoroughly establish that he entertained none. "The 5th and spinal nerves agree in these essential circumstances: they have all double origins; they have all ganglia on one of their roots; they go out laterally to certain divisions of the body; they do not interfere to unite the divisions of the frame; they are all muscular nerves, ordering the voluntary motions of the frame; they are all exquisitely sensible, and the source of the common sensibility of the surfaces of the body. When accurately represented on paper, they are seen to pervade every part; no part is without them; and yet they are symmetrical and simple as the nerves of the lower animals."*

* Phil. Trans. 1821.

We will not pursue the arguments of the "Old Pupil" any further. When, upon the principal points in discussion, so much weakness is shown, we may well be excused for not inflicting upon our readers a detailed answer to the less important statements contained in the above letter. If we discover, said Samuel Johnson, one part of a line to be of worsted, we do not expect the remainder to be of silk.

